# Effects of centrifugation and whole-body vibrations on blood–brain barrier permeability in mice

**DOI:** 10.1038/s41526-019-0094-z

**Published:** 2020-01-07

**Authors:** David Dubayle, Arnaud Vanden-Bossche, Mathieu Beraneck, Laurence Vico, Jean-Luc Morel

**Affiliations:** 1 Université Paris Descartes, Sorbonne Paris Cité, Paris, France; 2 CNRS, Integrative Neuroscience and Cognition Center, UMR 8002, Paris, France; 3 INSERM UMR 1059 - SAnté INgéniérie BIOlogie, Université de Lyon, St Etienne, France; 4 CNRS, Institut des Maladies Neurodégénératives, UMR 5293, Bordeaux, France; 5 Université de Bordeaux, Institut des Maladies Neurodégénératives, UMR 5293, Bordeaux, France

**Keywords:** Physiology, Neuroscience

## Abstract

Modifications of gravity levels induce generalized adaptation of mammalian physiology, including vascular, brain, muscle, bone and immunity functions. As a crucial interface between the vascular system and the brain, the blood–brain barrier (BBB) acts as a filter to protect neurons from pathogens and inflammation. Here we compare the effects of several protocols of hypergravity induced by centrifugation and whole-body vibrations (WBV) on BBB integrity. The immunohistochemistry revealed immunoglobulin G (IgG) extravasation from blood to hippocampal parenchyma of mice centrifuged at 2 × *g* during 1 or 50 days, whereas short exposures to higher hypergravity mimicking the profiles of spaceflight landing and take-off (short exposures to 5 × *g*) had no effects. These results suggest prolonged centrifugation (>1 days) at 2 × *g* induced a BBB leakage. Moreover, WBV were similarly tested. The short exposure to +2 × *g* vibrations (900 s/day at 90 Hz) repeated for 63 days induced IgG extravasation in hippocampal parenchyma, whereas the progressive increase of vibrations from +0.5 to +2 × *g* for 63 days was not able to affect the IgG crossing through the BBB. Overall, these results suggest that the BBB permeability is sensitive to prolonged external accelerations. In conclusion, we advise that the protocols of WBV and centrifugation, proposed as countermeasure to spaceflight, should be designed with progressively increasing exposure to reduce potential side effects on the BBB.

## Introduction

During spaceflights, organisms are exposed to confinement, radiations and successive modifications of gravity levels, including hypergravity (HG) during take-off and landing phases and microgravity during the orbital flight. Spaceflight affects the cardiovascular system via the blood shift responsible for the decreases in plasma volume and cardiac performance and probably the increase of intracranial blood pressure. In vertebrates, these gravity changes and alteration of blood pressure impact many sensory systems (vision; taste and olfaction; proprioception) but have prominent consequences on the vestibular system with major impact on balance, posture and spatial representation. Moreover, links between the vestibular system and the autonomous nervous system could be responsible for orthostatic intolerance induced by the decrease of baroreflex observed in astronauts after their return from space (all of these concepts were reviewed^1–4^). Exposition to artificial gravity during and/or after spaceflight by centrifugation has been proposed as a countermeasure to limit or suppress neurovestibular consequences of prolonged weightlessness such as the orthostatic intolerance observed after spaceflight.^5,6^ Likewise, whole-body vibrations (WBV) serves as a countermeasure against cardiovascular alterations due to aging and obesity, as it enhances vasodilation of small arterioles, and possibly capillaries in human leg muscles,^7,8^ and reduces the bone loss classically observed after spaceflight.^9,10^

The cardiovascular adaptation to gravity changes can alter the functions of endothelial and smooth muscle vascular cells.^11–14^ In the brain, these cells ensure the quality of the blood–brain barrier (BBB). Recently, it has been suggested in mice that the BBB could be altered after hindlimb unloading (a model reproducing microgravity-induced cardiovascular effects observed during spaceflights) in combination with radiations,^15^ but this phenomenon has not been investigated yet.

The most important function of the BBB is to assume strict controlled exchanges between blood and brain parenchyma, including glucose transport and clearance of toxic compounds, among which those suspected to be responsible for neurodegenerative disorders. BBB destabilization has been demonstrated to increase the incidence of stroke and neurodegenerative diseases as vascular dementia.^16,17^ Moreover, the breaking of the BBB has been implicated in the transfer of the malaria parasite into the brain.^18^

Immunoglobulin G (IgG) extravasation is commonly used as an index of BBB disruption.^19^ Because centrifugation and WBV are proposed as countermeasures to microgravity, we have tested several protocols of centrifugation and WBV on the BBB integrity by measuring the presence of immunoglobulins in the hippocampal parenchyma.

## Results

### HG but not WBV reduces the body weight increase and food intake

**Fig. 1:**
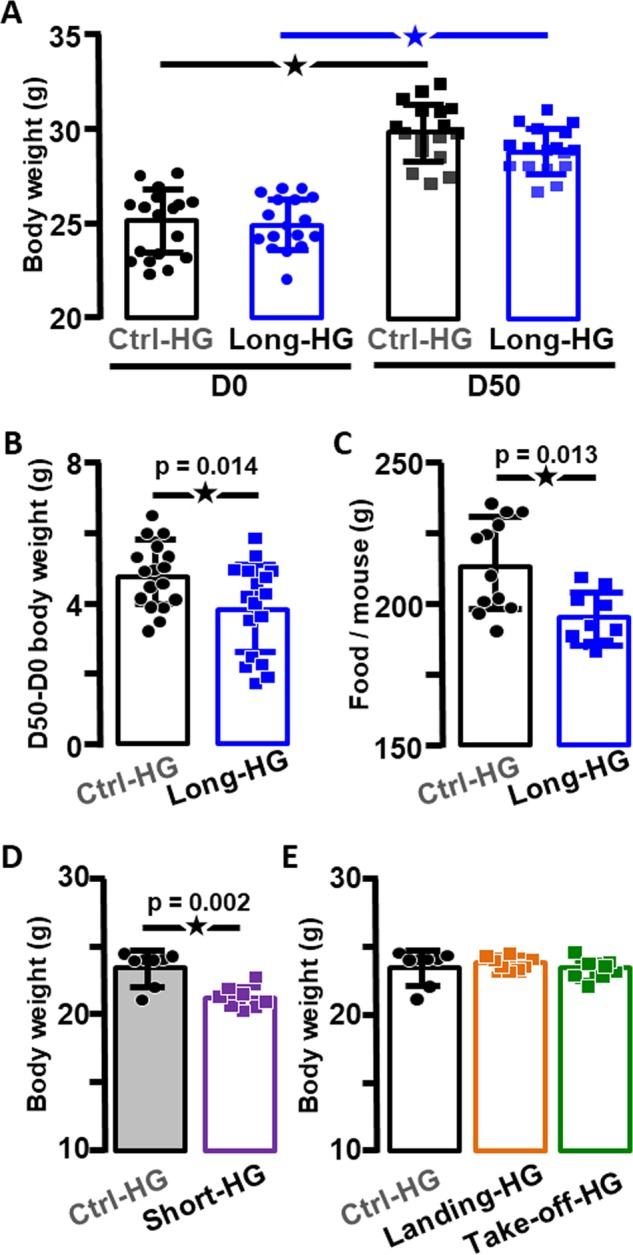
Alterations of body weight and food intake in hypergravity-exposed mice. **a** Body weight of control mice (Ctrl-HG) and mice exposed to long-term hypergravity (Long-HG), before (left panel) and at the end of the experiment (right panel, here stars indicate *p* < 0.001). **b** Difference of body weight after 50 days in the Ctrl-HG and Long-HG groups. **c** Total food intake in Ctrl-HG and Long-HG. **d** Body weight in Ctrl-HG and after short-term exposure to hypergravity (Short-HG); **e** body weight in Ctrl-HG and landing and take-off HG protocols.

The comparison by two-way analysis of variance (ANOVA) of the body weight of Ctrl-HG and Long-HG mice indicated that both groups presented similar weights before as well as just after the end of the centrifugation and that both groups increased their body mass (*p* < 0.0001, Fig. 1a). However, two-way ANOVA, followed by Sidak multiple comparisons tests, specifies that this increase in weight was significantly affected in the Long-HG group compared to controls (*p* = 0.021 for the interaction time × condition, this *p* value is not indicated in the graphs as it represents an interaction). The analysis of the differences between weights measured at both time points, expressing the increases of body weight (Delta weight), confirmed this last effect (Fig. 1b). As expected, the 50 days exposure to 2 × *g* reduced the increase of body weight. In addition, the total food intake during centrifugation (measured as the difference between the amount of food provided and food remaining) was significantly decreased by the 2 × *g* exposure (Fig. 1c). Likewise, the exposure to 2 × *g* for 24 h was also sufficient to decrease the body weight (Fig. 1d). In contrast, the exposure to higher gravity for shorter period during landing and take-off conditions did not influence the mouse body weight (Fig. 1e).

The exposure to 63 days to WBV did not have any effect on the body weight of mice (as already published in Gnyubkin et al.^9^), including the Short-WBV group (26.6 ± 0.37 × *g*, *n* = 8 versus 27.1 ± 0.59 × *g*, *n* = 8 for the Ctrl-WBV group, *p* = 0.48, not illustrated).

### HG induces the increase of BBB leakage

**Fig. 2:**
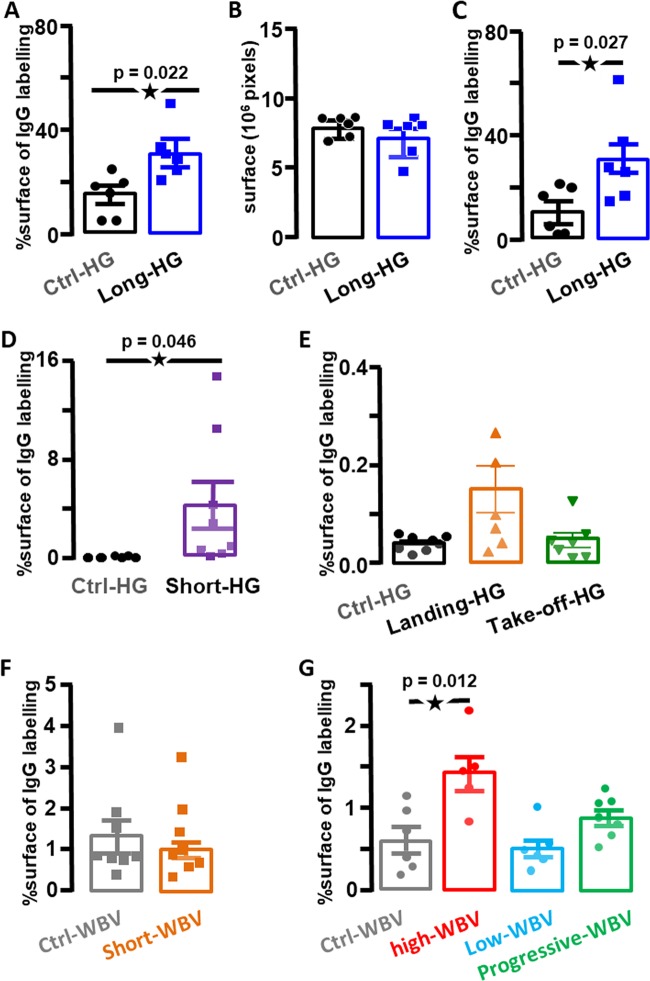
Effects of centrifugation and WBV on IgG extravasation in the hippocampus. **a** IgG extravasation expressed as the percentage of labelling observed in Long-HG and Ctrl-HG groups for the entire surface of hippocampus. **b** Comparison of the surfaces of hippocampus in Long-HG and Ctrl-HG groups. **c** IgG extravasation expressed as the percentage of labelling for the constant area of hippocampus in the Long-HG and Ctrl-HG groups. **d**, **e** IgG extravasation expressed as the percentage of labelling observed in the Short-HG, landing and take-off groups in comparison with their control. **f**, **g** IgG extravasation expressed as the percentage of labelling observed in the WBV groups in comparison with their control. Statistical significant differences are reported. The star indicates *p* < 0.05.

**Fig. 3:**
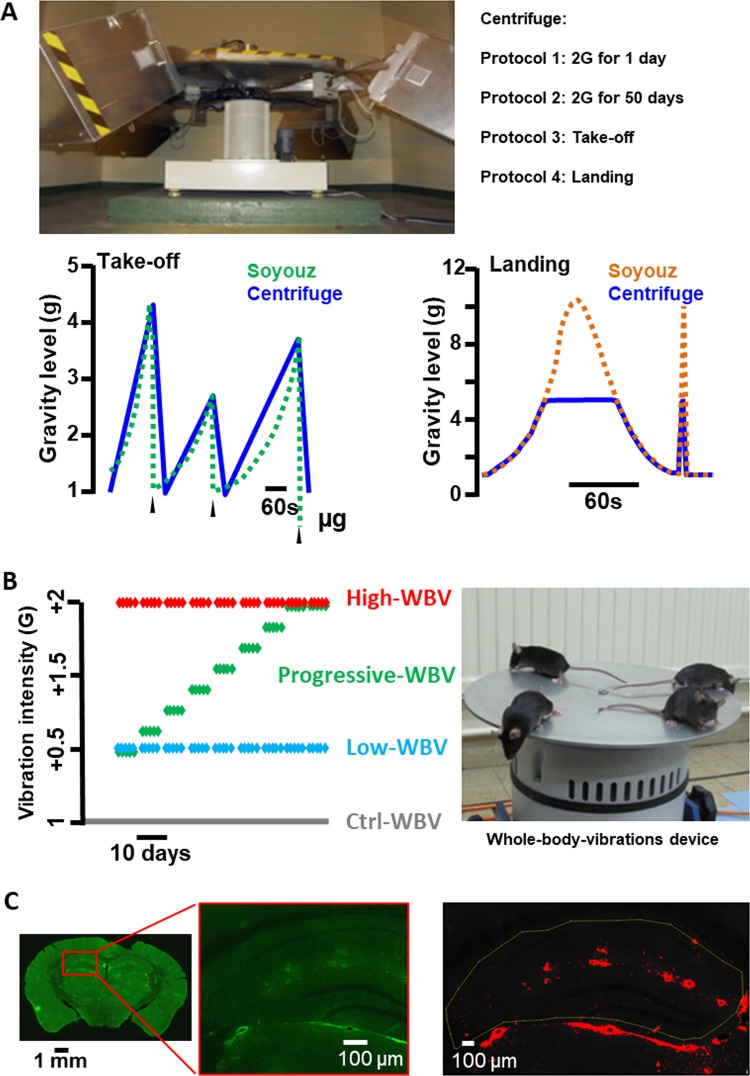
Hypergravity and whole-body vibration protocols. **a** Picture of the centrifuge. Bottom panels, profiles of gravity levels of the centrifuge reproducing those measured during landing and take-off of the Soyuz mission. **b** Vibration protocols of WBV (one dot = one day of vibration) and picture of mice on the device. **c** Example of the imaging process to interpret the immunostaining with, from left to right, typical imaging generated by Nanozoomer on one slide of brain (IgG coupled with FITC appears in green), the magnification of this image for fluorescence evaluation, and the image of a labelled area after application of the threshold on the analysed area (in red).

The exposure to HG during 50 days led to a large increase of the staining of IgG in the brain parenchyma (Fig. 2a), suggesting the BBB leakage. However, HG changes could also modify the ratio between ventricle and brain parenchyma, as expressed by a small decrease of the measured area of the hippocampus (Fig. 2b). Normalization of the IgG staining by the surface of hippocampus measured on each slice confirmed a significant increase in the BBB permeability (Fig. 2c). In another experiment, the increase in BBB leakage was similarly observed in the brain obtained from mice exposed to 2 × *g* for 24 h (Fig. 2d). In contrast, the higher HG levels (closed to 5 × *g*) and the iteration of accelerations (Fig. 3a) observed during landing and take-off conditions, respectively, did not affect significantly the presence of IgG in brain parenchyma (Fig. 2e).

### Long-term WBV also induce BBB leakage

Similarly, the BBB leakage was estimated after WBV protocols. First, single exposure to WBV at 2 × *g* was tested and revealed no change in BBB permeability (Fig. 2f). Then iterated exposure (63 consecutive days) to vibrations was tested following the protocols described in “Methods” with different intensities of vibrations. A significant increase of IgG was observed in the Long-WBV group exposed to 2 × *g* vibrations for 63 days, whereas the Low-WBV (0.5 × *g* vibrations for 63 days) as the Progressive-WBV (increasing *g* level for 63 days) did not show any significant increase in IgG labelling (Fig. 2g). This result suggests that the intensity (level of gravity) and duration of the vibrations critically influence the BBB leakage.

## Discussion

As previously shown, the increase of body weight was reduced after a long-lasting exposure to HG, which has been proposed as an index of the centrifugation.^20–22^ We have pinpointed that this reduction started as soon as the first day of centrifugation. One factor involved in this weight reduction could be the decrease of food intake provoked by vestibular over stimulation, as we recently demonstrated during short-term motion sickness protocols.^23^

In contrast, short but intense HG stimulation as during the take-off and landing of Soyuz, as well as single or repetitive WBV, did not have any influence on the mice body weight, suggesting that no major metabolic changes are induced in these cases and/or that episodic (<4 min) vestibular stimulation were not sufficient to trigger motion sickness-like symptoms.

The literature about effects of gravity change on BBB efficacy is more speculative than demonstrative. In the cardiovascular field, hindlimb unloading decreases the BBB efficacy only when it is associated with “space-like” radiations. This result suggests that simulated microgravity alone is not sufficient to alter the BBB.^15^ Concomitant effects of gravity changes and radiations were also observed on vascular reactivity in humans, as well as in the hindlimb unloading model,^12^ indicating that the phenotypic alterations induced by gravity changes were locked by radiation exposure. In other words, this work suggests that microgravity exposure modifies temporarily the cardiovascular reactivity but that the radiations, suffered during lunar voyages, can alter cardiovascular functions definitely. Until now, the BBB function had only been explored indirectly via the overexpression of Aquaporin-4, a vascular water transporter, and weeks after the end of hindlimb suspension.^15^ In our study, the leakage of IgG from blood to brain was assessed shortly after the centrifugation was stopped (maximum 2 h), confirming the direct alteration of the BBB function due to the HG protocol. If we consider that the long-term exposure to 2 × *g* induced a physiological adaptation to the new gravity level, then the stop of the centrifuge could be compared to a decrease of gravity level (as proposed in the reduced gravity paradigm^24^), and our results could confirm the previously described effects of microgravity on BBB. Moreover, if we consider that this leakage is also possible just after 24 h of exposure to 2 × *g*, we confirm that an alteration of the BBB could be a rapid, localized and reversible process, as recently reported during epileptic seizure.^25^ Finally, the BBB leakage induced by gravity changes could be temporary and this question should be addressed.

In mouse models as in humans, the WBV has been reported to increase bone and muscle recovery after traumatic injury or to reduce the impact of aging.^9,26^ Depending on the *g* level, HG has similar effects on mineral density of bone or muscle phenotypes.^10,27^ Our data on BBB opening and body weight show that the effects of daily applications of WBV were different to those observed after continuous centrifugation, suggesting different adaptive mechanisms in both conditions. Other experiments will be required to differentiate the effects of WBV and centrifugation on regulation of metabolism.

Alterations of the time–body weight curve are regularly proposed as an index of the stress level due to environmental changes. The role of stress on BBB function is controversial: several types of stress can induce an impairment of the BBB permeability,^28,29^ whereas glucocorticoids reinforce the BBB efficacy by increasing the synthesis of tight-junction proteins.^30^ Considering all the protocols of our study, we have tested several HG challenges varying in terms of intensity and duration. The daily and progressive application of WBV from +0.5 to +2 × *g* consisted in a habituation of mice to the protocol, which can be a way to effectively reduce the stress. On the other hand, exposure to centrifugation could be interpreted as a protocol involving mainly two phases of stress, i.e. when the mice in normogravity are first exposed to 2 × *g*, and symmetrically when they return to normogravity (as proposed in the reduced gravity paradigm). It is noticeable that continuous centrifugation and progressive WBV did not have the same effects on BBB leakage and body weight increases, revealing that both protocols do not affect similarly the physiological status of the mice.

The BBB integrity is preserved in mice exposed to the take-off and landing protocol, suggesting that the duration more than the intensity of HG or WBV can induce the BBB leakage. We hypothesize that at least 30 min to a couple of hours are required to induce the BBB leakage.^25^ Shorter durations as those used in take-off and landing experiments were not sufficient to induce BBB leakage even at high gravity intensity. This mechanism could be related to: (1) impairments of the vestibular and/or sympathetic regulations of cerebral blood flow and cerebrospinal fluid that can alter the tight junctions and their regulations in endothelial cells to reduce the resistance of the BBB via the modification of shear stress induced by adaptation to 2 × *g*, as proposed on endothelial cell lines^31,32^, or (2) a modification of the ratio between local blood pressure and cerebrospinal fluid, inducing pressure-evoked endothelial cell responses. Both hypotheses do not exclude each other and the molecular determinants as well as the kinetics of this change in the effectiveness of the BBB remain to be determined in vivo. Our study indicates new research pathways to reveal how vestibulo-sympathetic alteration, nature of stress, vascular status and metabolism might synergistically act on the BBB.

Finally, although the impairment in memory processes in people exposed to WBV are not clearly demonstrated,^32,33^ several studies in mice have suggested that alterations of spatial navigation and memory could be induced by gravity changes.^34^ This is supported by transcriptomic and proteomic analysis of brain in mice models,^22,35,36^ and more recently in humans by magnetic resonance imaging.^4,37–40^ As also proposed in the field of neurodegenerative disorders, an impairment of the BBB is suggested to induce or accelerate the processes of the memory impairments.^17^ Our work participates to confirm the link between BBB dysfunction induced by gravity changes and putative memory impairments hypothesized recently,^15^ yet this point should be investigated to understand its molecular basis. Moreover, the short exposure to HG seems to impact the BBB in 50% of the mouse population (Fig. 2d), suggesting that individual mice are not equally sensitive to gravity changes. A comparable result was previously shown for the adaptation of the vestibulo-ocular reflex of mice following exposure to long-term HG.^41^ The interindividual responses to gravity changes have been identified as a critical factor in space physiology and should be the next challenge in the space biology field.

In humans, exposures to vibrations are proposed as physiotherapeutic methods. Our results suggest that (1) centrifugation could “open the door” to toxic molecules through BBB leakage, (2) daily and progressive WBV could have fewer side effects than centrifugation on BBB opening and may be considered as a safer countermeasure after or during microgravity exposure or as a therapeutic application to restore bone mineralization and muscle mass, and (3) an exposure to centrifugation could be efficient to guide molecules into the brain, such as therapeutic antibodies.

## Methods

### Animals

The experimental protocols have been approved by the French ministry of research, the local ethical committee (CEEA-Loire) and the Animal Welfare Committee of the PLEXAN (Platform for Experiments and Analysis, Faculty of Medicine, Université de Saint-Etienne, France, agreement no. 180801) in accordance with the principles of the European community. A total of 72 male C57BL/6J mice (2–4 -month old, Charles River, France) were used in the study. The animals were housed under standard conditions (22 °C, humidity 55%; day/night cycle 12 h/12 h), with an unlimited access to food and water. The mice were familiarized with experimental rooms during the week preceding the experiments. To reduce the number of animals involved in basic research, this project used the brains of animals involved in other protocols after their euthanasia (long-term exposures to centrifugation and WBV).

### HG protocol

The exposure to HG was performed by centrifugation of the mice from minutes to days. The centrifuge is illustrated in Fig. 3a. Four mice per cage (cage size: 36 × 20 × 14 cm) were placed in the gondolas of the centrifuge. After 1 h of habituation in the gondola, the different protocols of centrifugation were started. Control mice maintained in normogravity (1 × *g*) were placed in cages located in a static position in the same room as the centrifuge and at the same time, in order to expose them to similar environmental conditions except for the centrifugation (normogravity condition, 1 × *g*). The acute and chronic brain effects of HG at 2 × g exposure were evaluated by a short (1 day) and a long (50 days) period of centrifugation; in both cases, the centrifuge reached 2 × *g* and came back to 1 × *g* in 40 s. The 50-day duration was chosen to match previous long-term studies performed in this centrifuge,^41^ while the level of HG of 2 × *g* was preferred because of its limited effect on stress as revealed by blood corticosterone measurements.^20^ To change water and food, the centrifuge was stopped 4 times every 10 days during 1 h. Finally, groups of mice were exposed to profiles of acceleration in order to mimic the gravity levels and duration observed during take-off and landing of the Soyuz shuttle (Fig. 3a bottom panels). These acceleration profiles were obtained from the BION M1 spaceflight and adapted to take in consideration the characteristics of the centrifuge device. After centrifugation, the animals are placed in a protective enclosure and dropped from 60 cm high in order to mimic the acceleration produced by the landing (close to 10 × *g*, verified with accelerometer placed in the box).

Mice were divided into five different groups as follows:

—Ctrl-HG (*n* = 8): controls, not exposed to HG.

—Long-HG (*n* = 8): centrifuged at 2 × *g* during 50 days.

—Short-HG (*n* = 8): centrifuged at 2 × *g* during 1 day.

—Take-off-HG (*n* = 8): centrifuged from 1 to 4.3 × *g* during 530 s.

—Landing-HG (*n* = 8): centrifuged from 5 to 1 × *g* during 55 s.

### Whole-body vibrations

WBV device (TIRA TV 52120, Fig. 3b) was previously described.^9^ It is a shaker with an aluminium table on top (30 cm diameter, 4 mm thickness), which was used to generate WBV of the mouse in the vertical plane. Vibration parameters are based on the formula: *a* = (2π*F*)^2^.*d* (*a*: acceleration in m/s^2^, *F*: frequency in Hz, *d*: displacement in m) (with 9.81 m/s^2^ = 1 × *g*). Control mice were placed at the same time on the shaker in the absence of vibration (switched ON but not moving, to expose the mice to similar environmental conditions). Vibrated mice were daily exposed to a single vibration session during 900 s at 90 Hz but in different gravity conditions (from +0.5 to +2 × *g*) by modifying *d* parameter and for a short (1 day) or a long (63 days) period. The 63-day exposure was chosen based on literature showing that it affects physiological parameters, including bone mineralization.^9^ A series of complementary experiments allowed for daily vibrations that gradually increased every 7 days from +0.5 to +2 × *g* over 63 days. The five groups were as follows:

—Ctrl-WBV (*n* = 8): controls, not exposed to WBV.

—Low-WBV (*n* = 8): vibrated at +0.5 × *g* during 63 days.

—Progressive-WBV (*n* = 8): vibrated from +0.5 to +2 × *g* over 63 days.

—Short-WBV (*n* = 8): vibrated at +2 × *g* for 1 session.

—Long-WBV (*n* = 8): vibrated at +2 × *g* during 63 days.

Brain samples were collected at the end of each HG or WBV protocols.

### Immunohistochemistry

Mice received a lethal injection with sodium pentobarbital (175 mg/kg, intraperitoneal) before performing intracardiac perfusion of 30 mL phosphate-buffered saline (PBS 0.01 M, pH: 7.4) followed by 30 mL of Formalin solution (10%, Merck, HT501128) to rinse the blood and to fix the tissues, respectively. The mice were all killed 2 h after stopping the centrifuge. The brain was removed by fine dissection, post-fixed at 4 °C during 24 h in Formalin solution and preserved at room temperature during 48 h in a 30% sucrose–PBS solution. Coronal sections of the brain (40-μm thickness) were made using a freezing microtome (Frigomobil Reichert-Jung) and incubated overnight with a goat antibody against mouse IgG directly coupled to fluorescein isothiocyanate (FITC; 115-095-003, Jackson laboratory, diluted 1:100). After the last wash in PBS-DAPI (4′,6-diamidino-2-phenylindole), the sections were mounted in Mowiol-based medium. Brain sections presenting any alteration in the cortex due to their manipulation during dissections were excluded, as the specific signal due to the fluorescence could be altered.

### Image acquisition and statistical analysis

Fluorescence of immunohistochemistry labelling was observed in the hippocampus by confocal microscopy (SP5, Leica Microsystems) and the slide scanner Nanozoomer 2.0HT (Hamamatsu Photonics). The acquisition parameters of the SP5 set-up were adjusted on non-labelled sections (without anti-mouse IgG antibody coupled to FITC) to optimize measurements^42^. Nanozoomer 2.0HT contains a fluorescence imaging module using objective UPS APO ×20 NA 0.75 combined to an additional lens ×1.75, leading to a final magnification of ×35. Virtual slides were acquired with a TDI-3CCD camera. Fluorescent acquisitions are done with a mercury lamp (LX2000 200 W—Hamamatsu Photonics, Massy, France) and the set of filters adapted for DAPI and FITC fluorescence (Fig. 3c). The focus was realized on the DAPI labelling of the nuclei. To evaluate the importance of labelling, its intensity was estimated by means of fluorescence. The total surface occupied by fluorescence in the hippocampus was determined per unit area (μm^2^) using the ImageJ software. Results were reported as the ratio of labelled surface on analysed surface expressed in percentage.

The data were statistically compared using paired *t* tests, non-parametric Mann–Whitney test or one- and two-way ANOVA with post hoc comparison when applicable using normogravity as the control condition. The software used was GraphPad Prism (La Jolla, CA), which calculated the *p* value as the probability to observe two identical conditions. If *p* < 0.05, the two compared conditions were considered statistically different.

### Reporting summary

Further information on experimental design is available in the Nature Research Reporting Summary linked to this paper.

## Supplementary information

Reporting Summary Checklist

## Data Availability

Data are available by request from the author for correspondence.

## References

[1] Evans, J. M., Knapp, C. F. & Goswami, N. Artificial gravity as a countermeasure to the cardiovascular deconditioning of spaceflight: gender perspectives. *Front. Physiol*. **9**, 10.3389/fphys.2018.00716 (2018). 10.3389/fphys.2018.00716 PMC6043777 30034341

[2] Hallgren, E. et al. Dysfunctional vestibular system causes a blood pressure drop in astronauts returning from space. *Sci. Rep*. **5**, 10.1038/srep17627 (2015). 10.1038/srep17627 PMC4680856 26671177

[3] Norsk P (2014). Blood pressure regulation IV: adaptive responses to weightlessness. Eur. J. Appl. Physiol..

[4] Van Ombergen A (2017). The effect of spaceflight and microgravity on the human brain. J. Neurol..

[5] Clément G, Paloski WH, Rittweger J, Linnarsson D, Bareille MP (2016). Centrifugation as a countermeasure during bed rest and dry immersion: what has been learned?. J. Musculoskelet. Neuronal Interact..

[6] Clément G, Pavy-Le Traon A (2004). Centrifugation as a countermeasure during actual and simulated microgravity: a review. Eur. J. Appl. Physiol..

[7] Figueroa A, Jaime SJ, Alvarez-Alvarado S (2016). Whole-body vibration as a potential countermeasure for dynapenia and arterial stiffness. Integr. Med. Res..

[8] Beijer Å, Degens H, Weber T, Rosenberger A, Gehlert S (2015). Microcirculation of skeletal muscle adapts differently to a resistive exercise intervention with and without superimposed whole-body vibrations. Clin. Physiol. Funct. Imaging.

[9] Gnyubkin V, Guignandon A, Laroche N, Vanden-Bossche A, Malaval L (2016). High-acceleration whole body vibration stimulates cortical bone accrual and increases bone mineral content in growing mice. J. Biomech..

[10] Gnyubkin V, Guignandon A, Laroche N, Vanden-Bossche A, Normand M (2015). Effects of chronic hypergravity: from adaptive to deleterious responses in growing mouse skeleton. J. Appl. Physiol..

[11] Dabertrand F, Porte Y, Macrez N, Morel J-L (2012). Spaceflight regulates ryanodine receptor subtype 1 in portal vein myocytes in the opposite way of hypertension. J. Appl. Physiol..

[12] Delp, M. D., Charvat, J. M., Limoli, C. L., Globus, R. K. & Ghosh, P. Apollo lunar astronauts show higher cardiovascular disease mortality: possible deep space radiation effects on the vascular endothelium. *Sci. Rep*. **6**, 10.1038/srep29901 (2016). 10.1038/srep29901 PMC4964660 27467019

[13] Morel JL (1997). Effect of a 14-day hindlimb suspension on cytosolic Ca2+ concentration in rat portal vein myocytes. Am. J. Physiol..

[14] Sofronova SI (2015). Spaceflight on the Bion-M1 biosatellite alters cerebral artery vasomotor and mechanical properties in mice. J. Appl. Physiol..

[15] Bellone, J. A., Gifford, P. S., Nishiyama, N. C., Hartman, R. E. & Mao, X. W. Long-term effects of simulated microgravity and/or chronic exposure to low-dose gamma radiation on behavior and blood–brain barrier integrity. *Npj Microgravity* **2**, 10.1038/npjmgrav.2016.19 (2016). 10.1038/npjmgrav.2016.19 PMC5516431 28725731

[16] Lee MR, Jayant RD (2019). Penetration of the blood-brain barrier by peripheral neuropeptides: new approaches to enhancing transport and endogenous expression. Cell Tissue Res..

[17] Sweeney MD, Zhao Z, Montagne A, Nelson AR, Zlokovic BV (2019). Blood-brain barrier: from physiology to disease and back. Physiol. Rev..

[18] Beghdadi W (2008). Inhibition of histamine-mediated signaling confers significant protection against severe malaria in mouse models of disease. J. Exp. Med..

[19] Pelegrí C (2007). Increased permeability of blood-brain barrier on the hippocampus of a murine model of senescence. Mech. Ageing Dev..

[20] Guéguinou N (2012). Stress response and humoral immune system alterations related to chronic hypergravity in mice. Psychoneuroendocrinology.

[21] Bojados M, Jamon M (2014). The long-term consequences of the exposure to increasing gravity levels on the muscular, vestibular and cognitive functions in adult mice. Behav. Brain Res..

[22] Pulga A, Porte Y, Morel J-L (2016). Changes in C57BL6 mouse hippocampal transcriptome induced by hypergravity mimic acute corticosterone-induced stress. Front. Mol. Neurosci..

[23] Idoux, E., Tagliabue, M. & Beraneck, M. No gain no pain: relations between vestibulo-ocular reflexes and motion sickness in mice. *Front. Neurol*. **9**, 918 (2018). 10.3389/fneur.2018.00918 PMC6240678 30483206

[24] Van Loon, J. J. W. A. Centrifuges for microgravity simulation. The reduced gravity paradigm. *Front. Astron. Space Sci*. 10.3389/fspas.2016.00021 (2016).

[25] Mendes NF (2019). The blood-brain barrier breakdown during acute phase of the pilocarpine model of epilepsy is dynamic and time-dependent. Front. Neurol..

[26] Marin-Puyalto J (2018). Is vibration training good for your bones? An overview of systematic reviews. Biomed. Res. Int..

[27] Mirzoev T (2017). Divergent anabolic signalling responses of murine soleus and tibialis anterior muscles to chronic 2g hypergravity. Sci. Rep..

[28] Friedman A (1996). Pyridostigmine brain penetration under stress enhances neuronal excitability and induces early immediate transcriptional response. Nat. Med..

[29] Sharma HS, Nyberg F, Cervos-Navarro J, Dey PK (1992). Histamine modulates heat stress-induced changes in blood-brain barrier permeability, cerebral blood flow, brain oedema and serotonin levels: an experimental study in conscious young rats. Neuroscience.

[30] Roszkowski M, Bohacek J (2016). Stress does not increase blood–brain barrier permeability in mice. J. Cereb. Blood Flow. Metab..

[31] Szulcek R, van Bezu J, Boonstra J, van Loon JJ, van Nieuw Amerongen GP (2015). Transient intervals of hyper-gravity enhance endothelial barrier integrity: impact of mechanical and gravitational forces measured electrically. PLoS ONE.

[32] Maier JA, Cialdai F, Monici M, Morbidelli L (2015). The impact of microgravity and hypergravity on endothelial cells. Biomed. Res. Int..

[33] Liu Q, Zhou R, Zhao X, Oei TPS (2015). Effects of prolonged head-down bed rest on working memory. Neuropsychiatr. Dis. Treat..

[34] Porte Y, Morel J-L (2012). Learning on Jupiter, learning on the Moon: the dark side of the G-force. Effects of gravity changes on neurovascular unit and modulation of learning and memory. Front. Behav. Neurosci..

[35] Wang T (2017). iTRAQ-based proteomics analysis of hippocampus in spatial memory deficiency rats induced by simulated microgravity. J. Proteomics.

[36] Wang Y (2016). Effect of prolonged simulated microgravity on metabolic proteins in rat hippocampus: steps toward safe space travel. J. Proteome Res..

[37] Lee JK (2019). Spaceflight-associated brain white matter microstructural changes and intracranial fluid redistribution. JAMA Neurol..

[38] Riascos RF (2019). Longitudinal analysis of quantitative brain mri in astronauts following microgravity exposure. J. Neuroimaging.

[39] Van Ombergen A (2018). Brain tissue–volume changes in cosmonauts. N. Engl. J. Med..

[40] Van Ombergen A (2019). Brain ventricular volume changes induced by long-duration spaceflight. Proc. Natl Acad. Sci..

[41] Beraneck M, Bojados M, Le Séac’h A, Jamon M, Vidal PP (2012). Ontogeny of mouse vestibulo-ocular reflex following genetic or environmental alteration of gravity sensing. PLoS ONE.

[42] Morel J-L, Fritz N, Lavie J-L, Mironneau J (2003). Crucial role of type 2 inositol 1,4,5-trisphosphate receptors for acetylcholine-induced Ca2+oscillations in vascular myocytes. Arterioscler. Thromb. Vasc. Biol.

